# Cytokeratin 18 in plasma of patients with gastrointestinal adenocarcinoma as a biomarker of tumour response

**DOI:** 10.1038/sj.bjc.6605175

**Published:** 2009-07-14

**Authors:** L C Scott, T R J Evans, J Cassidy, S Harden, J Paul, R Ullah, V O'Brien, R Brown

**Affiliations:** 1 Centre for Oncology and Applied Pharmacology, University of Glasgow, Cancer Research UK Beatson Laboratories, Garscube Estate, Switchback Road, Glasgow G61 1BD, UK; 2 Cancer Research UK Clinical Trials Unit, Level 0, The Beatson West of Scotland Cancer Centre, 1053 Great Western Road, Glasgow G12 0YN, UK; 3 Department of Oncology, Cyclotron Building, Hammersmith Hospital, Du Cane Road, London W12 0NN, UK

**Keywords:** cytokeratin 18, biomarkers, gastrointestinal adenocarcinomas, chemotherapy, apoptosis

## Abstract

**Background:**

Plasma biomarkers may be particularly useful as a predictor or early marker of clinical response to treatment in addition to radiological imaging. Cytokeratin 18 (CK18) is an epithelial-specific cytokeratin that undergoes cleavage by caspases during apoptosis. Measurement of caspase-cleaved (CK18–Asp396) or total cytokeratin 18 (CK18) from epithelial-derived tumours could be a simple, non-invasive way to monitor or predict responses to treatment.

**Methods:**

Soluble plasma CK18–Asp396 and CK18 were measured by ELISA from 73 patients with advanced gastrointestinal adenocarcinomas before treatment and during chemotherapy, as well as 100 healthy volunteers.

**Results:**

Both CK18–Asp396 and total CK18 plasma levels were significantly higher in patients compared with the healthy volunteers (*P*=0.015, *P*<0.001). The total CK18 baseline plasma levels before treatment were significantly higher (*P*=0.009) in patients who develop progressive disease than those who achieve partial response or stable disease and this correlation was confirmed in an independent validation set. The peak plasma levels of CK18 occurring in any cycle following treatment were also found to be associated with tumour response, but peak levels of CK18–Asp396 did not reach significance (*P*=0.01, and *P*=0.07, respectively).

**Conclusion:**

Plasma levels CK18 are a potential marker of tumour response in patients with advanced gastrointestinal malignancy.

Radiological imaging of tumours is an essential part of the practise of oncology, with a crucial role in screening programmes and in the diagnosis and staging of established disease. Furthermore, the assessment of tumour size by imaging, usually with computed tomography (CT) is a key component in determining tumour response in clinical practice. However, the development of a plasma biomarker to monitor treatment response would be advantageous in terms of ease of repeated analysis and use of resources compared with anatomical imaging. In addition, such biomarkers might give an earlier indication of potential response to treatment rather than the time lag, which is necessary to observe changes in tumour size. In patients with malignancy this may avoid administering toxic treatments with little prospect of benefit and hence start alternative treatments earlier.

Biomarkers are defined as characteristics that are objectively measured and evaluated as an indicator of normal biological processes, pathological processes, or pharmacological responses to a therapeutic intervention (Srivastava and Srivastava, 2005). The ideal marker has high sensitivity and specificity for diagnosis; its level should correlate with disease stage and response to treatment; and it should be easily and reproducibly measured. Unfortunately, the biomarkers currently available for use in the management of solid tumours do not fulfil all these criteria and, therefore, are not presently recommended for screening of the general population. At present the main uses of biomarkers are in determining prognosis, monitoring responses to treatment, and in detection of disease recurrence.

Carcinoembryonic antigen (CEA) is a member of a class of oncofetal antigens that are produced within the normal developing fetus, but only in minute amounts by normal adult cells. First described in 1965, it has become the most widely used biomarker in gastrointestinal malignancy (Hostetter *et al*, 1990) and can be measured quantitatively by an immunoradiometric assay in serum, but owing to its lack of sensitivity in the early stages of disease it is unsuitable for population screening. Although elevated levels were first observed in patients with colorectal cancer, it can also be raised in pancreatic, gastric, lung and breast cancer, and so is not specific to colorectal cancer. It can also be elevated in a number of benign conditions including cirrhosis, inflammatory bowel disease, pancreatitis, and up to 3% of healthy volunteers.

Studies performed looking at serum biomarkers in gastric adenocarcinoma have failed to provide a biomarker that is sensitive or specific enough for population screening (Byrne *et al*, 1990; Gaspar *et al*, 2001; Ucar *et al*, 2008). Biomarkers that have been studied include CEA, carbohydrate antigen 19-9 (CA19-9) and carbohydrate antigen 72-4 (CA72-4). CA19-9 is a monoclonal antibody raised against a colon carcinoma cell line to detect a monosialoganglioside in patients with gastrointestinal cancer (Koprowski *et al*, 1981). It is elevated in 20–40% patients with gastric cancer. The CA72-4 assay measures a tumour-associated glycoprotein (TAG-72) using two monoclonal antibodies. Raised serum TAG-72 levels have been observed in 33–59% patients with gastric cancer (Paterson *et al*, 1986; Heptner *et al*, 1989).

An assay that measures circulating soluble cytokeratin 19 (CK19), CYFRA 21-1, is based on two monoclonal antibodies to CK19 (Bodenmuller *et al*, 1994). CYFRA 21-1 has mostly been used clinically in lung, and head and neck cancers (Nisman *et al*, 1998; Yen *et al*, 1998). In patients with oesophageal cancer high preoperative levels of CYFRA 21-1 have been found to be associated with tumour progression and poor survival outcomes especially in patients with squamous cell carcinoma (Brockmann *et al*, 2000; Shimada *et al*, 2003). Serum squamous cell carcinoma antigen 2 (SCC-antigen) mRNA concentrations have been found to be associated with pathological changes in oesophageal cancer, but are not sensitive or specific enough to be used for screening (Yang *et al*, 2008).

At present CEA can be used to monitor treatment response in colorectal cancer in patients with elevated levels at baseline. However, there is no such biomarker available for the management of gastro-oesophageal cancer and it would be valuable to have a generic biomarker that could be applicable in routine clinical practise in monitoring treatment response in patients with both upper and lower gastrointestinal tract cancers receiving a range of different chemotherapy regimens.

Cytokeratin 18 (CK18) is a major component of the intermediate filament of simple epithelial cells and epithelial-derived tumours, and makes up approximately 5% of the total cell protein (Chou *et al*, 1993). It undergoes cleavage by caspases 3, 7, and 9 during apoptosis into proteolytic fragments (Caulin *et al*, 1997; Ku *et al*, 1997; MacFarlane *et al*, 2000). The monoclonal antibody, M30, recognises a neo-epitope of CK18 (CK18–Asp396 cleavage product) exposed after caspase-mediated cleavage during apoptosis, but not intact CK18 (Leers *et al*, 1999). A further ELISA (M65) uses two monoclonal antibodies specific for epitopes on CK18 to measure total (both caspase-cleaved and un-cleaved) soluble CK18. The two ELISAs can be used in conjunction to calculate the relative proportion of caspase-cleaved CK18 to total CK18 in plasma (Biven *et al*, 2003).

The objectives of this study were to compare plasma levels of caspase-cleaved and total soluble CK18 between healthy volunteers and patients with advanced gastrointestinal adenocarcinomas, and to determine if there was any correlation between changes in plasma cytokeratin 18 levels during palliative chemotherapy and response to treatment, and so determine if measurement of CK18 could potentially be used as a marker of clinical outcome.

We report here for the first time that plasma levels of caspase-cleaved and total CK18 are significantly higher in patients with gastrointestinal adenocarcinoma than in healthy volunteers, and that plasma CK18 levels before commencing treatment, and peak levels observed during treatment, may predict response to chemotherapy in patients with gastrointestinal adenocarcinomas.

## Materials and methods

### Patients with gastrointestinal adenocarcinomas

This was a single-centre, prospective, open, non-randomised study at the Beatson Oncology Centre, Glasgow, UK. Eligible patients were those with histologically or cytologically confirmed, locally advanced, or metastatic gastrointestinal adenocarcinoma who were due to start systemic anticancer therapy, with either (a) adenocarcinoma of the colon or rectum receiving fluoropyrimidine-based therapy; or (b) adenocarcinoma of the oesophagus or stomach receiving chemotherapy with a regimen containing a fluoropyrimidine and a platinum analogue. Inclusion criteria also included age ⩾18 years, ability to comply with study procedures, and life expectancy >3 months. Patients who had had systemic anticancer therapy or radiotherapy within the previous 6 weeks were excluded, as were women who were pregnant or lactating. Chemotherapy was administered according to local protocols. Disease response was assessed clinically and by computed tomography (CT) scans performed before commencing chemotherapy and at regular intervals during treatment (after cycles 3 and 6 for patients with gastric or oesophageal cancer and patients with colorectal cancer receiving capecitabine, and after cycles 6 and 12 in patients with colorectal cancer receiving infusional 5-flourouracil-based regimens). Disease response, as defined by the RECIST criteria (James *et al*, 1999), was determined from the radiology reports for individual patients. Epithelial toxicity experienced by patients during chemotherapy was assessed by review of an individual patient's case records, but detailed toxicity assessments were not prospectively recorded in this study. Sample collection from these patients commenced in November 2004 and is currently still ongoing. The study was approved by the West Glasgow Hospitals Research Ethics Committee, and all patients gave written informed consent before undertaking any study-related procedures.

Seventy-three patients with locally advanced or metastatic gastrointestinal adenocarcinomas were recruited in the initial exploratory phase, including 18 patients with oesophageal adenocarcinoma, 19 patients with gastric adenocarcinoma, and 36 patients with adenocarcinoma of the colon or rectum (Table 1). Thirty-two of the patients with gastric or oesophageal adenocarcinoma were treated with combination chemotherapy comprising epirubicin, cisplatin, and 5-FU (ECF), three were treated with a combination of cisplatin and 5-FU (CF), and two with carboplatin and 5-FU (CarboF). Thirty-three of the patients with colorectal cancer were treated with capecitabine monotherapy, and three with infusional 5-FU and folinic acid (modified ‘de Gramont’ regimen).

Baseline pretreatment plasma samples were collected from all patients undergoing chemotherapy. However, plasma was not available for all time points during treatment, highlighting the difficulties in the collection of clinical samples for this type of study; however, baseline pretreatment plasma samples were collected from all patients undergoing chemotherapy.

### Healthy volunteers

On one occasion, blood samples were collected from 100 healthy volunteers between November 2004 and February 2005. All volunteers completed a short health questionnaire that recorded current health status, previous medical history, family medical history, current medications, and smoking and alcohol intake history. The samples were collected anonymously and volunteers were not followed up subsequently and did not have any further health assessments as part of this study. This study was approved by the West Glasgow Hospitals Research Ethics Committee, and all volunteers gave written informed consent.

### Blood collection and preparation

Twenty milliliters of blood were collected into tubes containing EDTA (Greiner Bio-one Vacuette) before starting chemotherapy (baseline) and before administration of each subsequent course of chemotherapy (day 1) until discontinuation of systemic therapy. Additional 20 ml samples were collected at various time points during chemotherapy courses in selected patients, with the time points dependent on the specific chemotherapy regimen (day 2 for infusional 5-flourouracil regimens in colorectal cancer, and on days 2, 8, and 15 in patients with gastric or oesophageal cancer receiving continuous infusional 5-flourouracil). The time points for sample collection were chosen to coincide with patients’ scheduled appointments for treatment, which depended on the regimen received. Blood samples (20 ml) were collected into tubes containing EDTA (Greiner Bio-one Vacuette) on one occasion from the 100 healthy volunteers.

Plasma was separated within 2 h of collection by centrifuging the whole blood sample at 1500 **g** for 10 min at 20°C. The supernatant was then removed, placed in a separate 15 ml falcon tube and centrifuged again using the above conditions. The resulting supernatant was then aliquoted into Eppendorf tubes (Eppendorf AG, Hamburg, Germany) and immediately frozen at −70°C until analysis.

### Assay methods

Samples were assayed in duplicate for CK18–Asp196 using the M30-Apoptosense ELISA (PEVIVA AB, Bromma, Sweden), for which the normal range is ⩽180 U l^−1^ where 1 unit equals 1.24 pmol (manufacturer's brochure), and for total soluble CK18 using the M65-ELISA (PEVIVA) according to the manufacturer's instructions and the final result used for further analysis was the mean of the values from the duplicates. In brief, 25 μl of sample was added to each well, followed by 75 μl of HRP-conjugated monoclonal antibody. The samples were then incubated for 4 h at room temperature with constant shaking, after which excess unbound conjugate was removed by five washing steps. Colour development was then achieved by the addition of 200 μl of 3, 3,5,5′-tetramethyl-benzidine solution, followed by incubation for 20 min in the dark. The reaction was stopped by the addition of 50 μl of 1.0 M sulphuric acid and the absorbance measured in a microplate reader at 450 nm. Through plotting a standard curve of known concentrations of M30 antigen standards supplied in the kit against absorbance, the amount of antigen in the controls and unknown samples can be calculated by interpolation. Sample dilution was performed according to the manufacturer's instructions.

### Study design and sample size

The objectives of this study were to compare plasma levels of CK18–Asp196 and total soluble CK18 between healthy volunteers and patients with advanced gastrointestinal adenocarcinomas, to determine if there was any correlation between changes in plasma cytokeratin 18 levels during palliative chemotherapy and tumour response, and so determine if measurement of CK18 could potentially be used as a marker of clinical outcome. Other variables considered within the patient population included age, gender, disease extent at baseline, tumour marker (CEA) assessment (patient population only), epithelial toxicity and chemotherapy regimen received.

The sample size of 73 cancer patients was initially assessed in an exploratory study as this was the number of patient samples available. As the subsequent statistical analysis yielded positive correlations, a validation study was then performed using an additional 53 patients, a power analysis was performed to ensure that this number was adequate based on the associations observed in the exploratory study.

It was considered inappropriate to perform a survival analysis for this heterogenous group of patients with either advanced gastro-oesophageal or colorectal cancers receiving palliative chemotherapy with a number of chemotherapy regimens. Tumour response was considered a more robust indicator of clinical outcome for the purposes of this study.

### Statistical analysis methods

The distribution of the CK18, CK18–Asp396 and CEA values were markedly skewed so these variables were logged in all analyses. Where there were two classifying groups (e.g., gender) the Mann–Whitney *U*-test was used to compare these variables (or changes between variable values); when there were more than two groups (e.g., tumour site) the Kruskal–Wallis test was used. Spearman's rank correlation test was used when determining an association between either CK18 or CK18–Asp396 and age. The replicate variability in the assays was estimated from all the replicate results available in the study.

## Results

### Total soluble and caspase-cleaved CK18 levels: healthy volunteers

Plasma CK18–Asp396 levels within 100 normal healthy volunteers were assessed using the M30 Apoptosense ELISA. Within the population studied there was a wide range of values, from 51 to 849 U l^−1^, with a median of 121 U l^−1^. The values were independent of age (*P*=0.80) and gender (*P*=0.21). The range in female patients was 51–849 U l^−1^ and that in male patients was 79–616 U l^−1^. The total soluble CK18 levels were also determined for the 100 normal healthy volunteers using the M65 ELISA and as for the CK18–Asp396, there was a wide range in total soluble CK18 levels (161–899 U l^−1^), with a median of 312 U l^−1^. As for CK18–Asp396, the range in females was greater compared to the male patients (167–899 *vs* 161–630 U l^−1^). Again these values were not significantly associated with age (*P*=0.45) or gender (*P*=0.06).

### Total soluble and caspase-cleaved CK18: patients with gastrointestinal adenocarcinomas

**Figure 1:**
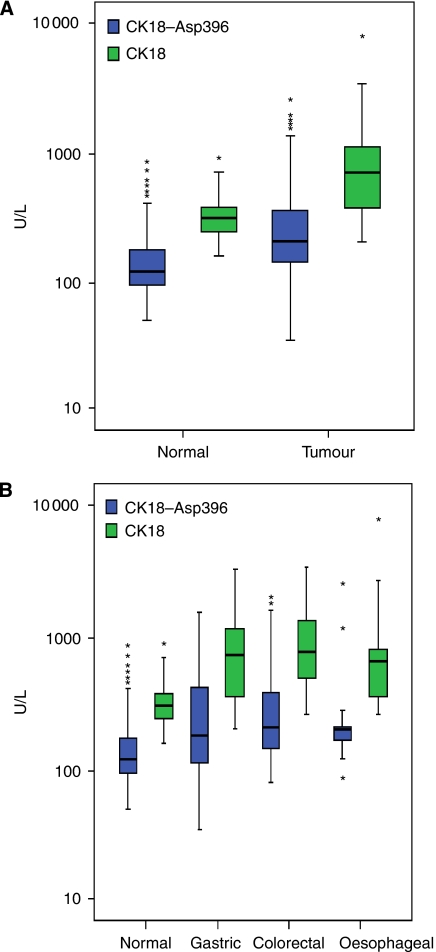
Plasma CK18–Asp396 and CK18 levels in patients *vs* healthy volunteers, and in different tumour types *vs* healthy volunteers. (**A**) Box plot demonstrating significantly higher plasma CK18–Asp396 and CK18 levels in patients with advanced gastrointestinal adenocarcinomas compared with healthy volunteers (*P*<0.001). (**B**) Box plot demonstrating significantly higher baseline CK18–Asp396 and CK18 plasma levels in patients with advanced esophageal (*P*<0.001, for CK18–Asp396 and CK18), colorectal (*P*<0.001 for CK18–Asp396 and CK18) and gastric (*P*=0.015, *P*<0.001, for CK18–Asp396 and CK18, respectively) cancer compared with healthy volunteers. U l^−1^ is defined according to the manufacturer's brochure where 1 unit equals 1.24 pmol. Outliers are shown by asterisk.

Baseline CK18–Asp396 and CK18 levels were measured in 73 patients with advanced gastrointestinal adenocarcinoma. The median CK18–Asp396 value was 207 U l^−1^ (range 35–2535 U l^−1^) and the median CK18 value was 717 U l^−1^ (range 206–7747 U l^−1^). The pretreatment plasma levels of both CK18–Asp396 and CK18 were significantly higher (*P*=0.015 for patients with gastric cancer and *P*<0.001 for patients with oesophageal and colorectal cancer) in the plasma samples of patients with all types of gastrointestinal adenocarcinoma examined compared with plasma samples from healthy volunteers (Figure 1 and Table 2).

**Figure 2:**
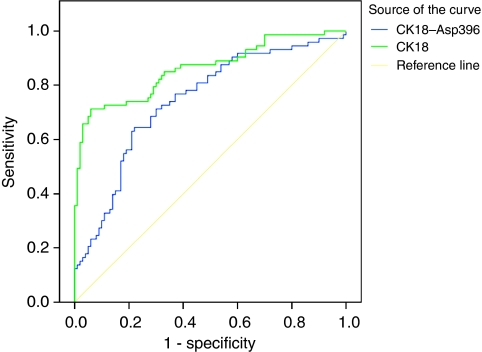
ROC curves for CK18–Asp396 and CK18 distinguishing between patients with advanced gastrointestinal malignancy and healthy volunteers. Outliers are shown by asterisk.

Receiver operating characteristic (ROC) curves for CK18–Asp396 and CK18 distinguished between patients with advanced gastrointestinal malignancy and healthy volunteers (Figure 2). Pooled data for all 73 patients demonstrated that CK18–Asp396 has a sensitivity of 27% at a specificity of 90% in distinguishing patients with advanced gastrointestinal malignancy and healthy volunteers. Similarly, CK18 has a sensitivity of 71% at a specificity of 90% in distinguishing between patients with advanced gastrointestinal malignancy and healthy volunteers. These results suggest that CK18 may be a better biomarker than CK18–Asp396 in distinguishing between plasma from patients with cancer and healthy volunteers, but that both markers may have limited use as a diagnostic marker.

Patients’ disease extent at baseline was associated with plasma CK18 and CK18–Asp396. Both the baseline plasma CK18–Asp396 and CK18 levels were significantly higher in the patients with metastatic disease compared with those with locally advanced disease; CK18–Asp396, median 210 U l^−1^ (range 58–2535 U l^−1^) *vs* 164 U l^−1^ (range 35–333 U l^−1^), CK18, median 833 U l^−1^ (range 260–7747 U l^−1^) *vs* 452 U l^−1^ (range 269–746 U l^−1^) (*P*=0.014 and *P*=0.011, for CK18–Asp396 and CK18, respectively).

### Correlation of plasma CK18 levels with tumour response

Objective tumour response data was available for 68 out of the 73 patients, and included 16 patients with partial responses, 25 patients with stable disease and 25 patients with progressive disease. A further two patients had rapid clinical disease progression which occurred before radiological disease assessment and were deemed to have progressive disease. Five others were non-evaluable as four had missing scan data and one patient was receiving adjuvant treatment. Patients’ case notes were also reviewed to document the timing and grade of epithelial toxicity observed during chemotherapy. This was then correlated with changes in plasma CK18 and CK18–Asp396.

**Figure 3:**
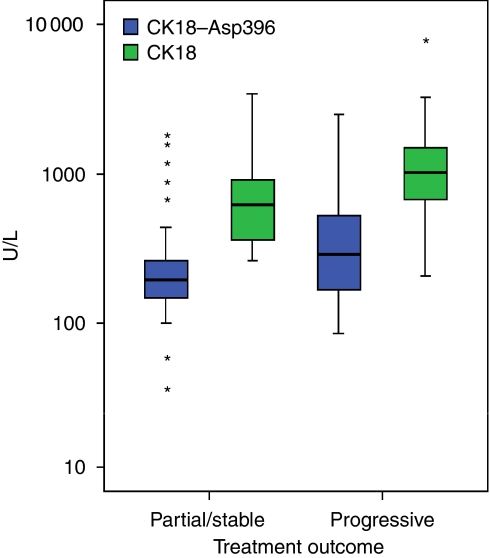
Baseline plasma levels of CK18–Asp396 and CK18 correlated with treatment outcome. Box plot demonstrating baseline CK18–Asp396 and CK18 plasma levels in patients who developed progressive disease through chemotherapy and partial response/stable disease. The CK18 level is significantly higher in patients with progressive disease (*P*=0.009, Mann–Whitney), (NE stands for non-evaluable treatment outcome). U l^−1^ is defined according to the manufacturer's brochure where 1 unit equals 1.24 pmol. Outliers are shown by asterisk.

The CK18–Asp396 and CK18 median plasma levels at baseline were higher (289 *vs* 194 U l^−1^ for CK18–Asp396, and 1021 *vs* 618 U l^−1^ for CK18) in patients who subsequently developed progressive disease during treatment (*n*=27) compared with patients who subsequently developed partial response or stable disease (*n*=41), although was only statistically significant for CK18 (*P*=0.125, *P*=0.009, for CK18–Asp396 and CK18, respectively, see Figure 3). There is an overall sensitivity of 22% at a specificity of 90% for CK18–Asp396 and an overall sensitivity of 19% at a specificity of 90% for CK18 baseline plasma levels in distinguishing between patients who will subsequently progress through chemotherapy and those who will have partial response/stable disease with treatment.

**Figure 4:**
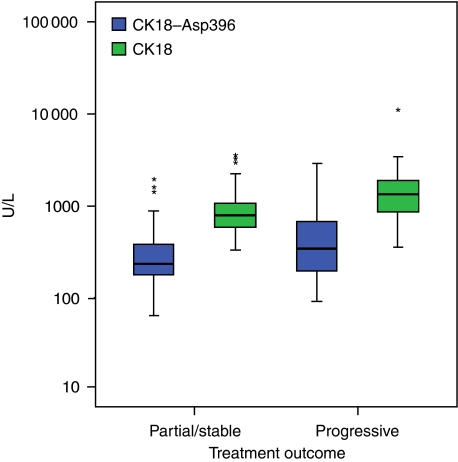
Peak plasma levels of CK18–Asp396 and CK18 correlated with clinical response (partial response/stable disease *vs* progressive disease). Box plot demonstrating peak plasma levels of CK18–Asp396 and CK18 in patients who developed progressive disease through chemotherapy compared with those who achieved a partial response/stable disease. The peak CK18 level is significantly with response (*P*=0.01). Outliers are shown by asterisk.

The plasma levels of CK18–Asp396 and CK18 were examined for each patient and the maximum level (or peak level) observed during treatment, defined as the maximum level that had been observed for each patient during any cycle of treatment, was compared with tumour response. Peak levels of CK18 were found to be associated with tumour response, but peak levels of CK18–Asp396 did not reach significance (*P*=0.01, and *P*=0.07, respectively: Figure 4A and B).

Comparison of the different chemotherapy regimens (ECF and capecitabine) and tumour response is confounded by comparing responses between the two different tumour groups (upper gastrointestinal cancer and colorectal cancer). Chi-squared test analysis of the relationship between the chemotherapy regimens and response (partial response/stable disease *vs* progressive disease) found no relationship between chemotherapy regimen and tumour response. Also, from the analysis the association between baseline plasma CK18–Asp396, total CK18 and response does not appear to be greatly affected when the chemotherapy regimen is allowed for.

Various patient demographic factors, including age, gender, and baseline disease extent (either locally advanced or metastatic disease at commencement of chemotherapy) were then analysed with treatment outcome to chemotherapy, but no correlation was found (*P*=0.514, *P*=0.149, and *P*=0.934, for age, gender, and disease extent, respectively). Similarly, there was no significant association between baseline plasma CK18–Asp396 and CK18 levels and patient's age (*P*=0.345, *P*=0.112 for CK18–Asp396 and CK18, respectively) and gender (*P*=0.519, *P*=0.257 for CK18–Asp396 and CK18, respectively). In addition, a straightforward visual inspection of the data revealed no obvious association between the timing of epithelial toxicity experienced and the occurrence of peak values of plasma CK18 and CK18–Asp396. However, the sampling for CK18 measurement was within the first 2 days of each cycle of treatment whereas epithelial toxicity is clinically observed mid-way through treatment cycles, although epithelial damage at the cellular level in normal tissues may well occur at the same time as in the cancer but become clinically apparent later during the treatment cycle.

In summary, plasma levels of CK18 at baseline are significantly higher in patients with progressive disease compared to patients with partial response/stable disease, and peak plasma levels of CK18 observed during treatment are associated with treatment response. Differences in plasma CK18–Asp396 and CK18 levels do not significantly associate with the patient's age, gender or the extent of disease at baseline.

### Validation study

A validation study was performed to see if baseline plasma levels of CK18–Asp396 and CK18 showed a reproducible correlation with treatment response in an additional cohort of 53 patients with advanced gastrointestinal malignancy. This included 25 patients with colorectal cancer, 15 with gastric cancer, and 13 with oesophageal cancer. Twenty-five of the patients with gastric or oesophageal adenocarcinoma were treated with combination chemotherapy comprising epirubicin, cisplatin, and 5-FU (ECF), one was treated with a combination of cisplatin and 5-FU (CF), one with carboplatin and 5-FU (CarboF) and one with epirubicin, carboplatin, and 5-FU (ECarboF). Fifteen of the patients with colorectal cancer were treated with capecitabine monotherapy, two with infusional 5-FU and folinic acid (modified ‘de Gramont’ regimen), five with combination chemotherapy comprising capecitabine and oxaliplatin, one with oxaliplatin and 5-FU (FOLFOX) and two with capecitabine, oxaliplatin, and cetuximab (see Table 3).

Objective tumour response data was available for all of the 53 patients, and included eight patients with partial responses, 24 patients with stable disease and 12 patients with progressive disease. A further nine patients had rapid clinical disease progression, which occurred before radiological disease assessment and were deemed to have progressive disease.

The validation group showed overlapping range and similar median values for CK18–Asp396 and CK18 as the original test set (median 158 U l^−1^, range 56–18786 U l^−1^ for CK18–Asp396 and median 660 U l^−1^ and range 235–19702 U l^−1^ for CK18). In the validation group, baseline plasma levels of both CK18–Asp396 and CK18 were again significantly associated with treatment outcomes (partial response/stable disease *vs* disease progression: *P*=0.028, *P*=0.003, respectively).

## Discussion

This is the first report, to our knowledge, documenting that the mean plasma pretreatment CK18 level is higher in patients with advanced gastrointestinal malignancy compared with healthy volunteers (Figure 1, Table 2). The groups were not age-matched, but levels of CK18 do not correlate with age. It is of note that the range of both plasma CK18–Asp396 and CK18 was wide in the healthy volunteers (Figure 1). Alcohol intake is known to increase caspase-cleaved CK18 values in serum as alcohol may cause apoptosis of liver cells (Natori *et al*, 2001). Other studies have also shown that viral illness, chronic hepatitis, and sepsis will increase levels of caspase-cleaved CK18 detected by the M30 Apoptosense ELISA kit (Bantel *et al*, 2004; Roth *et al*, 2004; Kronenberger *et al*, 2005). Thus the wide variation in caspase-cleaved CK18 values in healthy volunteers could possibly be due to intercurrent viral illness or alcohol consumption; however, the entry criteria for the healthy volunteer part of the study stated that they should have no significant past medical history, no evidence of intercurrent illness and no concomitant medications. A Mann–Whitney test was used to establish if an alcohol consumption of < or > 10 units per week affected the plasma CK18 levels in the healthy volunteers. The cutoff of 10 U per week was selected as this is below the recommended weekly consumption for both men and women. Within this group, alcohol consumption was not found to be associated with elevated plasma levels of either CK18–Asp396 or total soluble CK18 (*P*=0.91 and *P*=0.98, respectively).

The group of healthy volunteers was not followed up long-term and so it is unknown whether they subsequently developed any pathology to account for the variation in levels observed. As there is a significant overlap in plasma CK18–Asp396 levels between the healthy volunteers and cancer patients, it may be challenging to draw conclusions in individual cases.

After our sample collection from healthy volunteers had been completed, it was reported that elevated serum levels of caspase-cleaved CK18 may be an indicator of myocardial damage (Adlbrecht *et al*, 2007). However, healthy volunteers were ineligible if they had significant past or current illnesses. Although it was feasible that the patients with gastrointestinal cancer could have developed myocardial damage during the course of the study, these chemotherapy regimens are used with caution in patients with significant cardiac co-morbidities and we believe that it is unlikely that myocardial damage accounts for the levels of plasma CK18 observed.

We report in this study that the plasma CK18 level before commencing chemotherapy may predict treatment outcome in patients with advanced gastrointestinal adenocarcinoma. The patients with comparatively higher baseline levels of plasma CK18 tended to have higher levels of disease progression through chemotherapy compared to patients with lower baseline levels (Figure 3). This may be a reflection of the extent of disease present and potential for access to the circulatory system, as patients with metastatic disease had higher baseline levels of CK18 and CK18–Asp396 than those with locally advanced disease. However, when baseline disease extent as determined by CT scanning was correlated with treatment outcome, a statistically significant association was not found. This suggests that the baseline plasma CK18–Asp396 and CK18 levels may give an indication of both tumour burden and also the amount of cell death that is occurring, whether this is as a result of chemotherapy or as part of the ongoing disease process.

Pretreatment levels of CK18–Asp396 in the sera of patients with primary breast cancer, (*n*=152), recurrent breast cancer (*n*=49) or normal controls (*n*=82) (Ueno *et al*, 2003), demonstrated that patients with primary cancer had higher serum CK18–Asp396 levels than the normal controls (*P*=0.0001) and that patients with recurrent cancer had higher serum CK18–Asp396 levels than both the primary breast cancer patients and the normal controls (median values 180.5 *vs* 165.7 *vs* 156.2 U l^−1^ (*P*<0.0001 and *P*=0.008, respectively)). In addition, in patients with recurrent cancer the serum CK18–Asp396 level correlated with the number of involved organs, or burden of disease (*P*=0.041). In another study in patients with recurrent breast cancer (*n*=32) receiving chemotherapy with cyclophosphamide, epirubicin and 5-flourouracil or docetaxel (Biven *et al*, 2003), an index was calculated for each patient based on the difference between the maximum CK18–Asp396 level observed during treatment and the pretreatment level. Increases in serum CK18–Asp396 of at least 50% were significantly associated with clinical response (*P*=0.0001). More recently, the use of CK18 as a biomarker for monitoring chemotherapy-induced cell death in breast cancer has been published (Olofsson *et al*, 2007). In this study, both CK18–Asp396 and total CK18 were assessed using the ELISAs and drug-induced release of CK18 examined from breast carcinoma cells and tissue. Serum CK18 levels were then determined in 61 patients with breast cancer receiving either docetaxel or cyclophosphamide/epirubicin/5-flourouracil (CEF) chemotherapy. The results showed that CK18–Asp396 was released from cell and tissue cultures to the extracellular compartment and that the protein complexes were stable, suggesting that CK18-based assays would be applicable in clinical studies. In patients with breast cancer, docetaxel was found to induce increased levels of CK18–Asp396, indicating that the primary mode of cell death was apoptosis. In contrast, CEF induced increased levels of total CK18, indicating that the primary mode of cell death in these patients was necrosis. Also, the level of increase of total CK18 at 24hrs post-treatment correlated with clinical response to CEF (*P*<0.0001). Recently published studies of patients with hormone refractory prostate cancer (*n*=82) receiving palliative chemotherapy (Kramer *et al*, 2006) show significant increases in CK18–Asp396 usually between days 5 and 7 of each treatment cycle.

Two studies were recently published evaluating CK18 in patients with colorectal cancer. The first studied pre- and post-operative serum levels of CK18–Asp396 in 31 patients (Ausch *et al*, 2009). It also assessed serum levels of CK18–Asp396 in 10 patients receiving combination chemotherapy with oxaliplatin/capecitabine. The results showed that peri-operative levels of CK18–Asp 396 correlated significantly with tumour recurrence (*P*=0.016), but that increases in CK18–Asp 396 observed during chemotherapy did not correlate with response. The second study measured pre- and post-operative plasma levels of CK18–Asp396 and total CK18 in 49 patients with colorectal cancer and correlated the levels with patient and tumour characteristics, and survival outcomes (Koelink *et al*, 2009). The results showed that peri-operative plasma levels of both CK18–Asp396 and total CK18 were correlated with disease stage and were predictive of disease-free survival independent of tumour stage. Also the ratio of plasma CK18–Asp396/total CK18 which decreased with tumour progression, was also predictive of disease-free survival.

CK18–Asp 396 may also be applicable as a pharmacodynamic biomarker in phase I clinical trials of novel non-cytotoxic molecularly targeted anticancer therapies, with which objective tumour responses, as determined by a reduction in tumour dimensions by conventional imaging techniques, may not be observed. For example, our group has previously demonstrated that disease stabilisation was associated with CK18–Asp 396 plasma levels in patients with advanced solid tumours treated in a phase 1 clinical trial of the novel hydroxamate histone deacetylase inhibitor, belinostat (Steele *et al*, 2008).

It is of note that since our study started several papers have been published regarding sample-handling protocols for determination of serum and plasma CK18. Delays of greater than 4 h in sample acquisition and processing resulted in a significant increase in CK18–Asp396 levels. This effect was minimised by incubating the sample on ice. Furthermore, prolonged storage (>6 months) at −80°C resulted in higher levels of CK18–Asp396. The recommendation was that serum rather than plasma should be used to decrease the variation between the duplicate assays (Greystoke *et al*, 2008). Although plasma was used in our study, we had a robust standard operating protocol for sample acquisition that ensured all samples were processed and frozen to −80°C within 2 h of venesection. Other studies published have reported the stability of both CK18–Asp396 and total soluble CK18 in plasma from cancer patients stored at −80°C for over 2 years (Cummings *et al*, 2007). The plasma used in our study had been kept at −80°C for <2 years and care was taken to use samples that had not been repeatedly freeze thawed.

In conclusion, the results from our study suggest that measuring baseline and peak plasma levels of CK18 in patients receiving palliative chemotherapy for advanced gastrointestinal malignancy may help predict individual outcomes to therapy. However, a larger prospective clinical study is required to validate these results.

## Figures and Tables

**Table 1:** Table summarising patient demographic data

**Characteristics**	**Number of patients**
*Age*	
Median – 68 years	73
Range – 24–88 years	
	
*Gender*	
Male	41
Female	32
	
*Primary tumour site*	
Colorectal	36
Oesophageal	18
Gastric	19
	
*Disease extent*	
Locally advanced	11
Metastatic	60
Unknown/adjuvant	2
	
*Chemotherapy*	
Capecitabine	33
Modified de Gramont	3
ECF	32
CF	3
CarboF	2
	
*Chemotherapy cycles*	
Median – 4	
Range – 1–12	
	
*Clinical outcome*	
Partial response	16
Stable disease	25
Disease progression	27
Non evaluable	5

ECF=epirubicin/cisplatin/5-fluorouracil, CF=cisplatin/5-fluorouracil, CarboF=carboplatin/5-fluorouracil.

**Table 2:** Table summarising the median and range of CK18–Asp396 and CK18 plasma levels in healthy volunteers and in patients with gastrointestinal adenocarcinoma

	**CK18–Asp396 median value and range (U/l)**	**CK18 median value and range (U/l)**	***P*-value for CK18–Asp396 between tumour and healthy volunteers**	***P*-value for CK18- between tumour and healthy volunteers**
Healthy volunteers	121 (51–849)	312 (161–899)	—	—
Gastric cancer	183 (35–1569)	746 (206–3313)	*P*=0.015	*P*<0.001
Oesophageal cancer	204 (86–2535)	672 (266–7747)	*P*<0.001	*P*<0.001
Colorectal cancer	213 (81–1936)	780 (267–3482)	*P*<0.001	*P*<0.001

**Table 3:** Table summarising the patient demographic data in the validation study

**Characteristics**	**Number of patients**
*Age*	
Median 70 years	
Range 41–86 years	
	
*Gender*	
Male	31
Female	22
	
*Primary tumour site*	
Colorectal	25
Oesophageal	13
Gastric	15
	
*Disease extent*	
Locally advanced	14
Metastatic	39
	
*Chemotherapy*	
Capecitabine	15
Modified de Gramont	2
XELOX+/−cetuximab	2
FOLFOX	1
ECF	25
CF	1
Carbo F	1
ECarboF	1
	
*Chemotherapy cycles*	
Median 2	
Range 1–12	
	
*Clinical outcome*	
Partial response	8
Stable disease	24
Disease progression	21

XELOX=oxaliplatin/capecitabine; FOLFOX=oxaliplatin/infusional 5-flourouracil; ECF=epirubicin/cisplatin/5-flourouracil; CF=cisplatin/5-flourouracil; CarboF=carboplatin/5-flourouracil; EcarboF=epirubicin/carboplatin/5-flourouracil.
